# Neuroprotective effect of riboflavin kinase on cerebral ischemia injury in rats

**DOI:** 10.1186/s10020-025-01170-0

**Published:** 2025-04-02

**Authors:** Yingxin Zou, Minghua Ruan, Xu Feng, Fei Liu, Weihong Liu, Song Chen, Zhiyong Chu

**Affiliations:** Naval Medical Center, Naval Medical University, Shanghai, China

**Keywords:** Riboflavin kinase, Ischemic brain damage, Endoplasmic reticulum stress, Riboflavin

## Abstract

**Background:**

Riboflavin kinase (RFK, also called flavokinase) is a catalytic enzyme that converts riboflavin to its active form in vivo. Dysfunction of the RFK gene has been associated with susceptibility to ischemic stroke. However, the protective role and mechanisms of RFK in ischemic stroke have not been elucidated.

**Methods:**

Lentivirus-mediated RFK knock-up (RFK( +)) and knock-down (RFK(-)) were used to investigate the protective effect and mechanism of RFK in the rat middle cerebral artery occlusion (MCAO) model in vivo and in the oxygen and glucose deprivation (OGD) model of neurons in vitro; and the dependence of the protective effect of RFK on flavins was also investigated.

**Results:**

We demonstrated that RFK was an endogenous protein against ischemia brain injury both in vivo and in vitro experiments. RFK inhibited cerebral infarction, cerebral edema and neuronal apoptosis after cerebral ischemia. Its mechanisms include inhibition of the protein expression of Caspase 12 and Caspase 3 induced by cerebral ischemia, and thus inhibiting endoplasmic reticulum stress (ERS) and neuronal apoptosis; the protective effect of RFK depends on the presence of the flavoprotein Ero1; exogenous riboflavin supplementation protected cortical neurons from ischemic injury and prolonged the lifespan in stroke-prone spontaneously hypertensive rats with low RFK gene function, but this protective effect is limited and cannot completely reverse the decreasing trend of neuronal tolerance to ischemic injury caused by RFK gene dysfunction; the protective effect of RFK against ischemic injury is independent of the presence of flavins and their concentrations.

**Conclusions:**

The present study demonstrates that RFK is an important regulatory molecule against ischemia brain injury and its mechanism involves inhibition of ERS. The protective effect of RFK is independent of the presence of flavins and their concentrations. RFK deserves further investigation as a promising target gene for the detection of stroke susceptibility. Flavins may be used as a preventive or adjunctive treatments for ischemic brain injury.

**Graphical Abstract:**

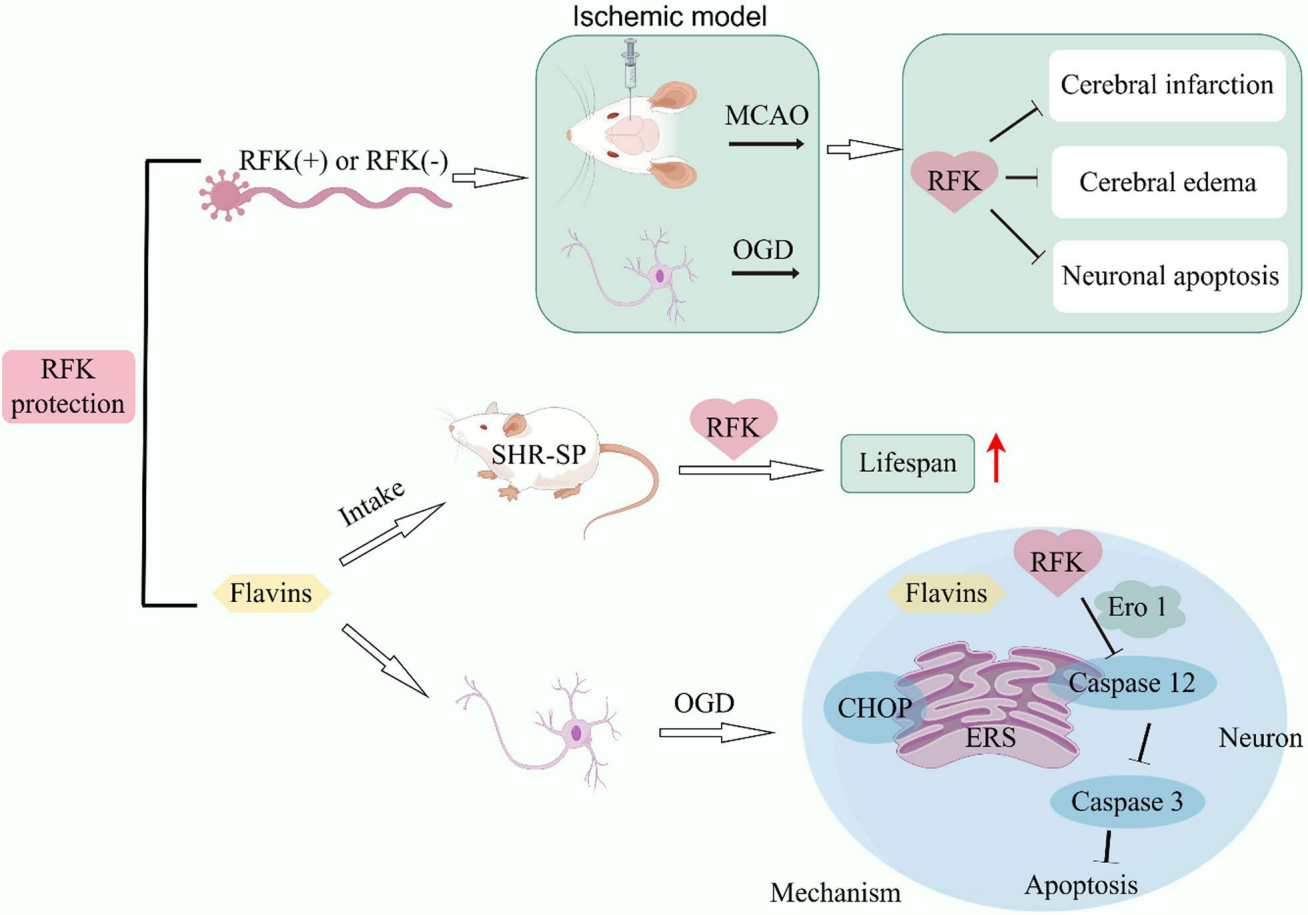

**Supplementary Information:**

The online version contains supplementary material available at 10.1186/s10020-025-01170-0.

## Introduction

Stroke, defined as localized cerebral dysfunction caused by acute cerebrovascular disease, is one of the most common cerebrovascular diseases worldwide, with high rates of death and disability (Hilkens et al. 2024). Stroke includes both ischemic stroke and hemorrhagic stroke. Ischemic stroke, also known as cerebral infarction, includes cerebral thrombosis and cerebral embolism and accounts for 85% of strokes (Maida et al. 2020; Kuriakose et al. 2020). Many experimental studies have been conducted to explore intervention targets in ischemic stroke (Zhou et al. 2024; Wang et al. 2024). However, few effective therapies or strategies have been established in clinical practice.

Our previous research found that susceptibility to ischemic stroke may be linked to the gene dysfunction of riboflavin kinase (RFK, also known as flavokinase (Merrill et al. 1980)) (Zou et al. 2012). RFK phosphorylates riboflavin to flavin mononucleotide (FMN), which is then rapidly adenylated to form flavin adenine dinucleotide (FAD) (Kanazawa et al. 2018). It is the rate-limiting step for riboflavin utilization in the body. Little is known about RFK in mammals. RFK variants have been reported to be associated with susceptibility to neurodegenerative diseases (Mehrpour et al. 2020). RFK has been reported to couple TNF receptor 1 to NADPH oxidase (Yazdanpanah et al. 2009). RFK binds and activates inducible nitric oxide synthase to participate in the regulation of macrophage polarization (Shan et al. 2024). However, the specific mechanism of the role of RFK in ischemic stroke remains to be investigated.

In the present study, we intensively investigated the protective role and its mechanisms of RFK against ischemic stroke. Gene knock-up and knock-down techniques were used to observe the effect of RFK on ischemic brain injury both in vivo and in vitro in rats; whether the protective effect of RFK is independent of flavins (including riboflavin, FMN and FAD) was also observed. Taken together, our results suggest that RFK inhibit ischemic brain damages, and thus may be explored as a promising preventive and therapeutic target for stroke.

## Materials and methods

### Animals

Wistar rats,196 in total, were provided by Sino-British Sippr/Bk Lab Animal Co., Ltd. Stroke-prone spontaneously hypertensive rats (SHR-SP), 30 in total, were kindly provided by Department of Pharmacology, School of Pharmacy, Naval Medical University. All rats were housed in cages of 5 at a temperature of 23 ± 3 °C and relative humidity of 55 ± 15%. Light was provided for 10 h per day and air was changed 20 times per hour. Food and water were provided on a free-access basis. Prior to the experiments, the rats were quarantined for 7 days.

### RFK(+)/RFK(−) lentivirus preparation

The RFK knock-up (RFK( +)) and RFK knock-down (RFK(−)) fragments were amplified and constructed into pLenti6.3-IRES2-EGFP /V5 DEST and pLenti6.3-MCS /V5 DEST series lentiviral expression vectors, respectively. The Lentivirus was then packaged and concentrated in 293 T cells. The lentivirus concentration was determined by fluorescence microscope observation method. Empty lentivirus concentration was 1.18 × 10^9^TU/ml; RFK( +) lentivirus concentration was 1.06 × 10^9^TU/ml; RFK(−) lentivirus concentration was 2.09 × 10^9^TU/ml. All lentiviruses were then uniformly diluted to 4 × 10^8^TU/ml.

### Lentivirus administration

We first investigated whether RFK expression could be changed by cerebral administration the lentivirus encoding RFK( +) or RFK(−).

**Fig. 1:**
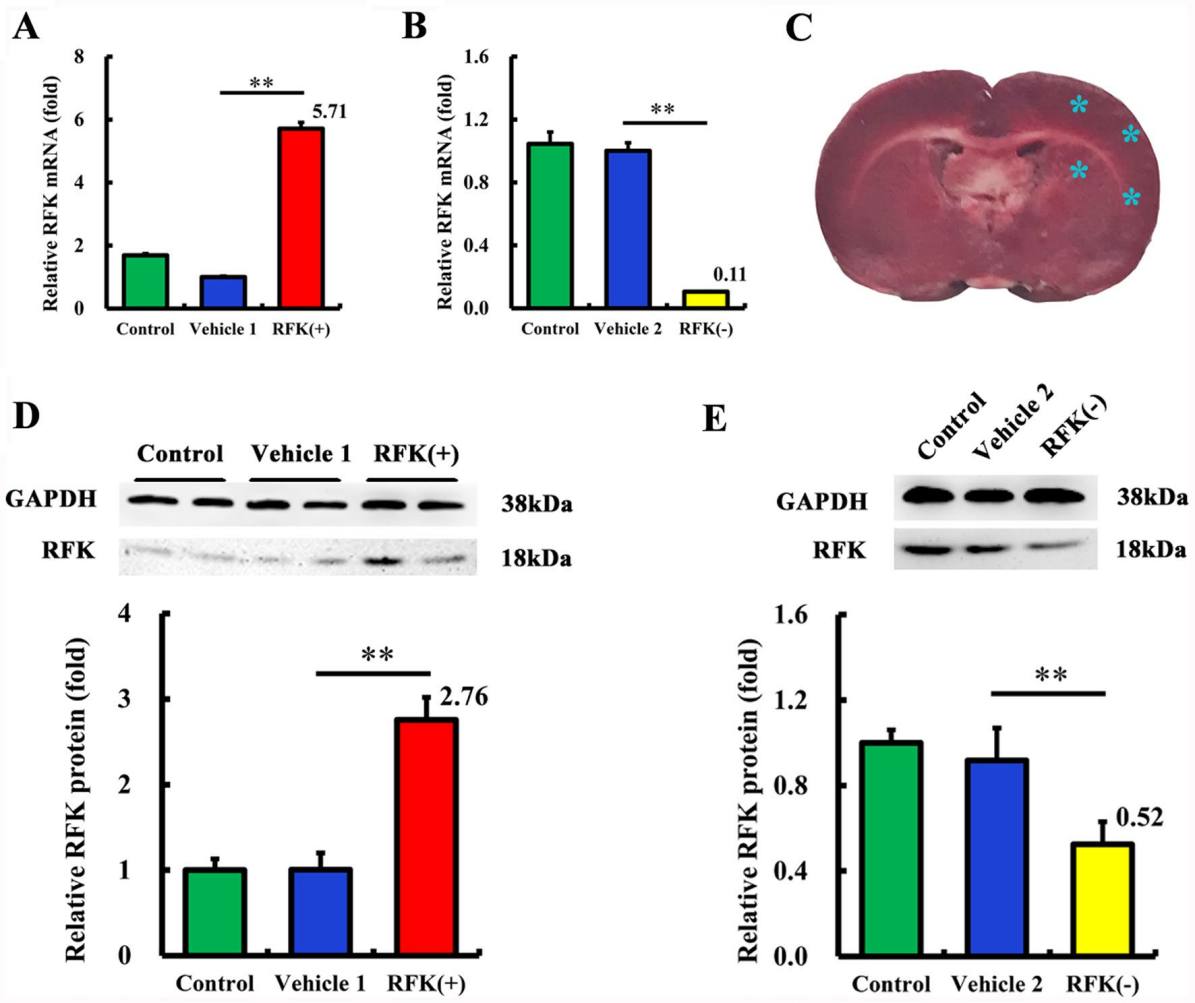
The RFK level is successfully regulated by the infection of lentivirus encoding RFK knock-up (RFK (+)) or RFK knock-down (RFK(-)). **A**–**B** mRNA levels of RFK in neurons. Neurons were infected with Lentivirus encoding RFK( +) or (RFK(−) (MOI = 10) for 12 h. After removal of the lentivirus and culturing for a further 3 days, the neuronal mRNA was extracted and detected by real-time PCR (n = 3). **C** Cerebral administration sites. **D**–**E** Protein levels of RFK in rat brains. Lentivirus encoding RFK (+), RFK (−), empty lentivirus (Vehicle) or saline control were stereotaxically administrated into the cortex and hippocampus at 4 sites (5 μl per site; 4 × 10^5^ TU/μl) in all. Three weeks later, the rats’ brains were dissected to extract protein. The protein levels were examined by Western Blotting (n = 6 or 3). **P < 0.01 compared with the vehicle group

Wistar rats, weighted 210 ± 10 g, were randomly arranged into the groups of control, model, vehicle, RFK( +) and RFK(−). Lentivirus infection was performed by cerebral administration the lentivirus encoding RFK( +), RFK(−) or empty vehicle into the brain of rats after anesthetizing with 3% pentobarbital sodium solution. Lentiviral vectors were injected into three sites located in the left cortex and one site located in the left hippocampus with 5 μl (4 × 10^5^ TU/μl) per site (Fig. 1C) for each rat. A stereotaxic apparatus (ASI Instruments, Houston, TX) was used to locate the injection sites. Location of the four sites: site 1, 0.3 mm anterior to bregma, lateral 3 mm to midline and depth 2 mm to skull surface; site 2, 0.3 mm anterior to bregma, lateral 3 mm to midline and depth 5 mm to skull surface; site 3, 0.3 mm anterior to bregma, lateral 5 mm to midline and depth 3 mm to skull surface; site 4, 0.3 mm anterior to bregma, lateral 5 mm to midline and depth 6 mm to skull surface.

After administrated infection for 21 days, MCAO was performed in the left brain of rat. After another 24 h, the rats were then sacrificed to dissect brain tissues for the following multiple detections.

### Middle cerebral artery occlusion (MCAO)

MCAO is an animal model that closely mimics the human ischemic stroke process and induces necrosis of the surrounding cerebral ischemic tissue.

Wistar rats, weighing 210 ± 10 g, were subjected to permanent MCAO which was performed by the intraluminal filament technique as described in previous reports (Tabet et al. 2020; Guo et al. 2013). The rats were anesthetized with 3% pentobarbital sodium solution. Cerebral focal ischemia was produced by intraluminal occlusion of the left middle cerebral artery using a silicone rubber-coated nylon mono-filament (2432-A4, Beijing Xinong Technology Co., Ltd). Sham-operated model rats underwent the same surgical procedure. After the surgery for another 24 h, the brain tissues were dissected and weighed to observed edema, and performed 1% 2,3,5-triphenyltetrazolium chloride (TTC) (Sinopharm Chemical Reagent Co. Ltd., Shanghai, China) staining to observe cerebral infarction as described (Guo et al. 2013). The degree of cerebral oedema was detected by MCAO lateral hemibrain weights.

### Neurons culture and transfection

Primary cultured cortical neurons from 16 to 18 day Wistar rat embryos were prepared according to previously established protocols (Zou et al. 2012). Briefly, neurons were isolated from the cerebral cortex of Wistar rat embryos. The cortex was dissected under a microscope. After removal of the meninges, the cortex was digested with 0.25% trypsin (GIBCO). Cells were cultured on poly-D-lysine-coated plates at a density of 1 × 10^6^ cells/ml in medium containing neurobasal medium (GIBCO) supplemented with 2% B27, 100 U/ml penicillin, 100 μg/ml streptomycin and 0.5 mM glutamine (GIBCO) and incubated at 37 °C with 5% CO_2_. In our laboratory, the cerebral cortex of a rat embryo is capable of producing between 12 × 10^6^ and 18 × 10^6^ cells. Medium was freshened twice a week.

Neurons were transfected with lentivirus encoding RFK( +) or RFK(-) for 12 h, and then continue to cultivate with fresh culture medium for 2–3 days.

### Oxygen and glucose deprivation (OGD)

The OGD model simulates glucose deprivation and hypoxia of neurons after stroke at the cellular level in vitro. On the day of the experiment, the neurobasal medium was removed. Neurons were washed twice with phosphate-buffered saline, transferred to high-glucose DMEM and incubated for 2 h in an incubator (gassed at 37 °C under 5% CO_2_ and 95% air). To model ischemia-like conditions in vitro, neurons were then exposed to the OGD culture medium (glucose-free DMEM) and placed in an incubator gassed with hypoxia gas (94% N_2_, 5% CO_2_ and 1% O_2_) at 37 °C for 1.5 h. The O_2_ concentration was mainteined at less than 1% by continuous flow of OGD gas through the incubator. Neuronal viability and protein expression were measured immediately after OGD. Neurons in the control group were treated in the same way without OGD.

### Neurons viability assay

The neurons viability analysis was performed using a 3-(4,5-dimethylthiazol-2-yl)-2,5- diphenyltetrazolium bromide (MTT) assay. The neurons were seeded in 96-well plates and cultured. The MTT dye (5 mg/mL) was added at 20 μl per well and the neurons were subsequently incubated at 37 °C for 4 h. The dark blue formazan crystals that formed in intact neurons were solubilized by 150 μl of dimethylsulphoxide (DMSO) per well. The absorbance was measured at 490 nm.

### Electron microscope assay

The brain tissues were cut into small pieces of 0.5 mm^3^; fixed in 0.1 M phosphate buffer containing 4% paraformaldehyde for more than 4 h; soaked in 0.1 M phosphate buffer for more than 4 h and replaced the buffer solution 3 times during this time; fixed in 0.1 M phosphate buffer containing 1% osmic acid; dehydrated gradually with alcohol and acetone; soaked in acetone and epoxy resin (Epon 812) 1:1 for 1 h, 1:2 for 5–8 h, and pure epoxy resin for 1 h; embedded in epoxy resin and aggregated in an oven; cut thin slices with a thickness of about 1.5–2 μm using a tissue slicer, and the desired area was located under a light microscope; sliced using an LKB ultra-thin sectioning machine, with a thickness of 70–80 nm, and collected on a 200–400 mesh copper mesh. The ultrathin sections were double stained with saturated uranyl acetate and lead citrate solution. Observed and photographed under a Hitachi H-7000 electron microscope.

### Flow cytometry assay

Apoptosis and death of cultured neurons induced by OGD were assessed using a commercial Annexin V-FITC/PI apoptosis detection kit (Keygen, Nanjing, China). After OGD, neurons were digested with 0.25% trypsin (GIBCO), collected and processed into a single-cell suspension. Neurons were then stained according to the manufacturer's protocols. Flow cytometry was performed on a Beckton Dickinson FAC-Scan and analyzed using CellQuest Pro software (BD, Franklin Lakes, NJ, USA). The green fluorescence of Annexin V-FITC was detected by excitation light at 488 nm and emission light at 530 nm; the red fluorescence of PI was detected by excitation light at 488 nm and emission light at 633 nm.

### Real-time PCR

The mRNA level of RFK was quantified by real-time PCR (Li et al. 2021a, b). Total RNA was extracted using Trizol (Invitrogen, Carlsbad, CA, USA), and 2 μg RNA was reverse transcribed into cDNA using RNA reverse transcriptase (Promega, Madison, WI, USA). Quantitative real-time PCR was performed in an Opticon Monitor 3 Real-Time PCR System (Bio-Rad, Hercules, CA, USA) on an ABI 7500 PCR instrument (Applied Biosystems, Carlsbad, CA, USA) using SYBR Premix Ex TaqTM Mixture (Takara, Otsu, Japan) according to the manufacturer's instructions. Primers for RFK were as follows 5'-GTGTCCACTGGCATCTATTACG-3' (sense) and 5'-CAGGTCTGAGGTAGCCAACAAT-3' (antisense); for GAPDH they were as follows: 5'-AGACCTCTATGCCAACACAGTGC-3' (sense) and 5'-GAGCCACCAATCCACACAGAGT-3' (antisense). GAPDH was used as an internal control. Real-time PCR was performed with a transcription efficiency of > 90%. The result was analyzed using the 2^−ΔΔCt^ method.

### Western blot

Western blot analysis was performed as we previously described (Zou et al. 2012). Briefly, total protein was immediately extracted and subjected to 12% SDS-PAGE (sodium dodecyl sulphate–polyacrylamide gel electrophoresis). Proteins were electro transferred to nitrocellulose membranes, incubated with primary antibodies for 2.5 h, and then incubated with the appropriate secondary antibodies. Images were captured and analyzed using the Odyssey infrared fluorescence imaging system (Li-Cor Bioscience, Lincoln, NE, USA).

Anti-Ero1L (1:1000, Abcam, ab81959); Anti-Caspase 12 (1:1000, Abcam, ab62484); Anti-Caspase 3 (1:1000, Cell Signaling Technology, 9665); Anti-CHOP (1: 1000, Cell Signaling Technology, 2895); Anti-RFK (1:1000, Abcam, ab133974); IRDye secondary antibodies (1:10,000, ROCKLAND, 611–131-003 and 610–131-003) were used for Western blotting.

### Lifespan assay

A total of 30 male SHR-SP were randomly arranged into 2 groups: control and riboflavin. The rats in the riboflavin group were fed with water supplemented with riboflavin at the final concentration of 120 mg/L until natural death. The rats in the control group were fed with water. The lifespans of the rats were recorded and the lifespan curve was drawn.

### Effect of flavins on OGD feurons

Neurons were infected with empty lentivirus (vehicle), RFK(+) lentivirus or RFK(−) lentivirus for 12 h (MOI = 10). After removing the lentivirus and culturing for a further 3 days, the neurons were subjected to the OGD program in the culture medium with or without flavins. The neurons viability was examined.

### Effect of erodoxin on OGD neurons

Erodoxin is the inhibitor of endoplasmic reticulum oxidase 1 (Ero1). Neurons were infected and cultured the same procedure as above. Neurons were then proceeded OGD program in the culture medium containing 25 nM Erodoxin. The neurons viability was examined.

### Statistical analysis

Data are expressed as mean ± SD. For data measurement, the Bartlett test was used to assess whether the variances were standard normal distributions. Subsequently, the F-test (ANOVA, two-sided) was performed to evaluate the variances. Fischer’s least significant difference (LSD) was used if the variances were homogenous; otherwise, Dunnett’s T3 test was used. For non-standard normal distributions, the Kruskal–Wallis test was used. If the result was notable, nonparametric tests were used to compare every two groups. The Log-rank test was used to evaluate the equality of survival curves. P < 0.05 was considered to indicate statistically significant difference.

## Results

### The RFK level is successfully regulated

We first examined the expression levels of RFK after regulation. The mRNA levels of RFK in neurons infected with RFK( +) or RFK(−) lentivirus were 5.71 and 0.11 times than those in the vehicle group, respectively (*P* < 0.01) (Fig. 1A, B). The protein levels of RFK in rat brains administrated with RFK( +) or RFK(−) lentivirus were 2.76 and 0.52 times than those in the vehicle group, respectively (*P* < 0.01) (Fig. 1D, E). These results indicated that, both in neurons in vitro and in rat brains in vivo, the RFK expression was successfully upregulated or downregulated by RFK( +) or RFK(−) lentivirus.

### RFK inhibits cerebral ischemic injury in rats

**Fig. 2:**
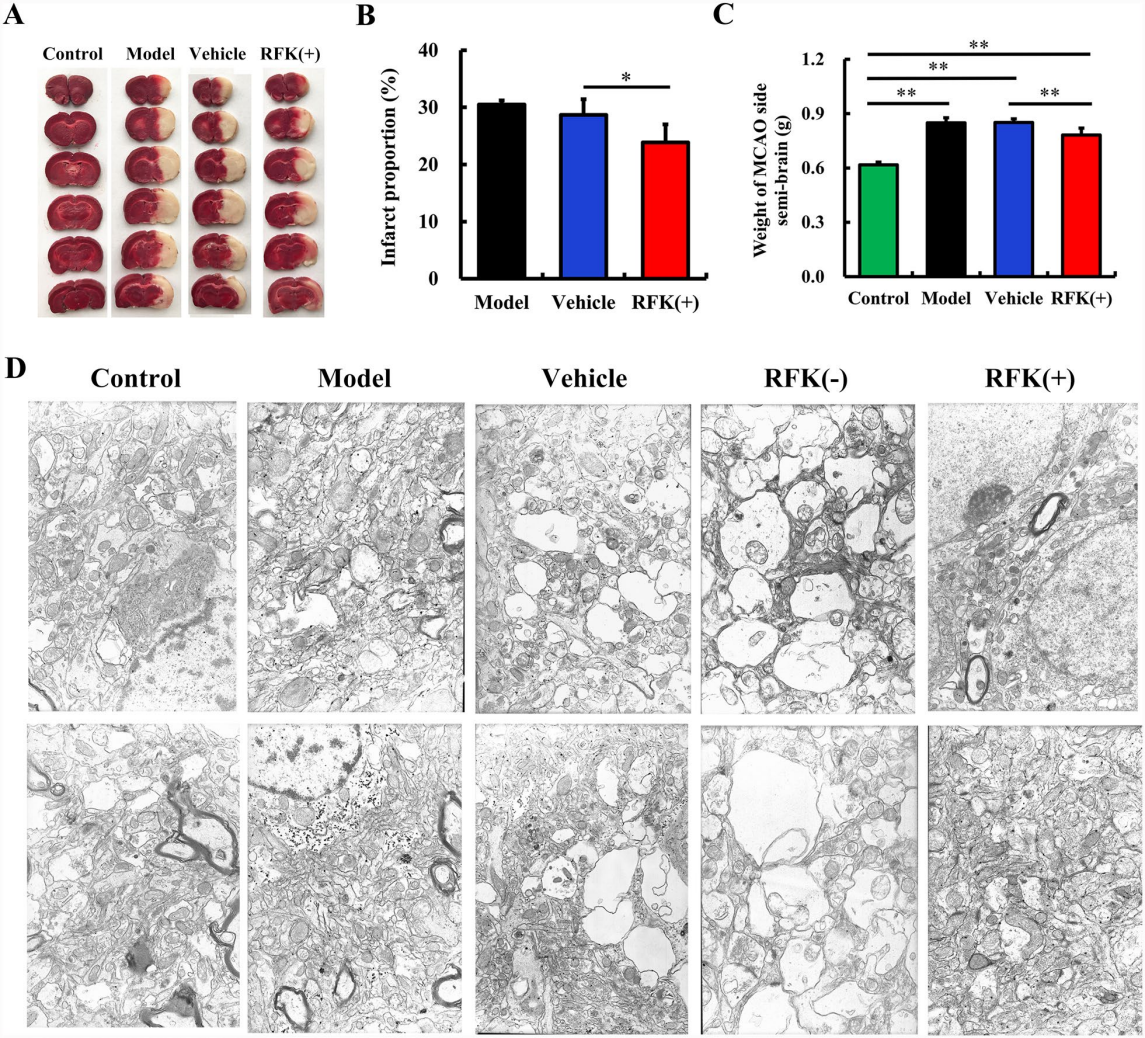
RFK inhibits the cerebral ischemic injury induced by middle cerebral artery occlusion (MCAO) in rats. Lentivirus encoding RFK (+), RFK (−), empty lentivirus (Vehicle) or saline control were stereotaxically administrated into the cortex and hippocampus at 4 sites (5 μl per site; 4 × 10^5^TU/μl) in all. Three weeks later, the rats underwent MCAO surgery. 24 h later, the rats’ brains were dissected for further examinations. **A** TTC-stained rat brain sections. **B** Infarct area statistics (n = 6). **C** Edema of the MCAO side brain (n = 6). **D** Ultrastructure of cortex (10,000 ×) (n = 3). Data are presented as mean ± SD. **P < 0.01; *P < 0.05

We next assessed the protective effect of RFK in rat brain. Rats were subjected to MCAO after cerebral administration of RFK( +) or RFK(−) lentivirus. As shown in Fig. 2, the infarct size (P < 0.05) (Fig. 2A, B) and the brain edema (P < 0.01) (Fig. 2C) induced by MCAO were significantly reduced in the RFK( +) group. We also observed the effect of RFK( +) and RFK (−) on the ultrastructural damage of cortical cells in rats after MCAO surgery. As shown in Fig. 2D, the nucleus of neurons in the control group is large and round, with abundant cytoplasmic contents and clear mitochondrial ridges; in the model group, mitochondria in neurons swelled, synaptic vesicles slightly expanded, some contents disappeared, and endoplasmic reticulum (ER) slightly expanded; in the vehicle group, synaptic vesicles in neurons expanded, contents disappeared, ER expanded and mitochondria had vacuoles; in the RFK(-) group, neurons swelled, synaptic vesicles in cells expanded extremely, contents disappeared, ER expanded and mitochondria extremely swelled; the structure of neurons in the RFK( +) group was basically normal and this state was similar to that of neurons in the control group. These results indicated that overexpression of RFK inhibited MCAO induced cortical injuries, while low expression of RFK aggravated MCAO induced cortical injuries. Conclusively, RFK inhibited the cerebral ischemic injuries in rats.

### RFK confers neuroprotection in vitro

We also monitored the protective effect of RFK in OGD model neurons in vitro. Neurons were divided into control, model, vehicle, RFK(+) and RFK(−) groups. The neurons were infected with lentivirus encoding RFK(+), RFK(−) or empty lentivirus (vehicle) for 12 h and then cultured for another 3 days. After 2 h of OGD model treatment, neuronal viability and apoptosis were determined by MTT method and flow cytometry, respectively.

**Fig. 3:**
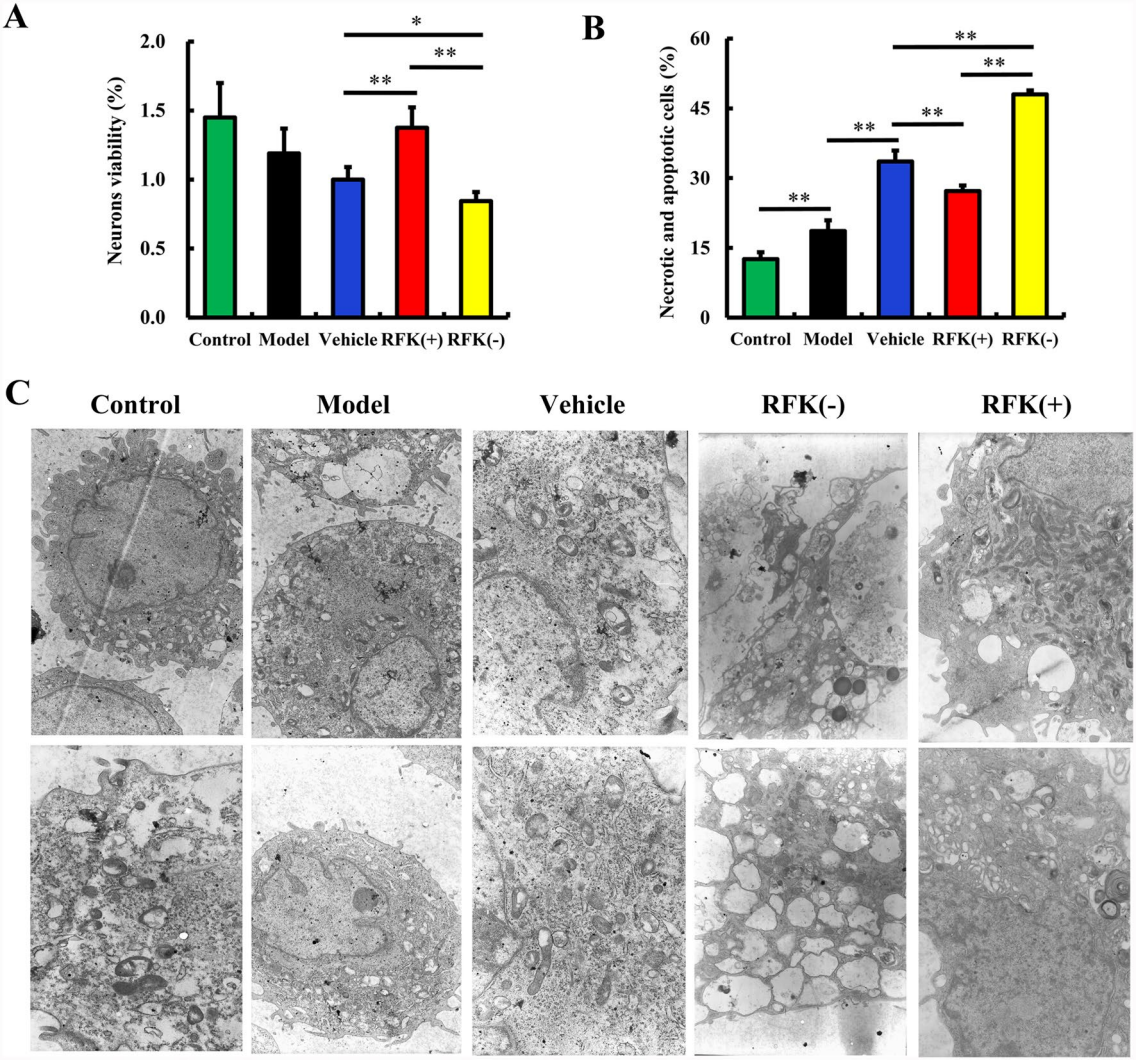
RFK inhibited oxygen and glucose deprivation (OGD) induced neuronal damages. Neurons were infected with empty lentivirus (vehicle), RFK knock-up (RFK (+)) lentivirus and RFK knock-down (RFK (−)) lentivirus for 12 h (MOI = 10). After removal of the lentivirus and culturing for a further 3 days, the neurons were subjected to the OGD program. Neurons viability was assessed by the MTT method. Neuronal necrotic and apoptotic rates were assessed by flow cytometry. **A** Neurons viability after OGD (n = 6). **B** Neuronal necrotic and apoptotic rates (n = 3). **C** Ultrastructure of neurons (10,000 ×) (n = 3). Data are presented as mean ± SD. *P < 0.05, **P < 0.01

As shown in Fig. 3A, compared to vehicle group, the viability of RFK (+) neurons was upregulated (P < 0.01), whereas the viability of RFK(-) neurons was downregulated (P < 0.05). This result suggested that RFK(+) protected neurons from OGD induced injury and RFK(−) exacerbated the injury. This was the evidence that RFK is a neuron protector in vitro.

Apoptosis after cerebral ischemia is responsible for an important part of ischemic brain damage (Sairanen et al. 2006). As shown in Fig. 3B and Additional file 1, compared with the control group, the neuronal necrotic and apoptotic rate significantly increased in the OGD model group; the infection of empty lentivirus in vehicle group further increased the neuronal necrotic and apoptotic rate after OGD; compared with the vehicle group, the neuronal necrotic and apoptotic rate was significantly decreased in the RFK( +) group (P < 0.01); while it was significantly increased in the RFK(-) group (P < 0.01). These results suggested that RFK(+) inhibited OGD induced neuronal apoptosis and death and thus has a protective effect; RFK(−) aggravated neuronal necrosis and apoptosis induced by OGD. This is consistent with the results of the neuronal activity assay described above and further confirms that RFK is a protective regulator against ischemic neuronal injury.

We also observed the effect of RFK on the neuronal ultrastructural damages induced by OGD. As shown in Fig. 3C, the nucleus of neurons in the control group is large and round, with abundant cytoplasmic content and clear mitochondrial ridges; mild expansion of synaptic vesicles was found in the neurons in the model group; in the vehicle group, neurons swelled, mitochondria swelled, some mitochondrial membranes ruptured, synaptic vesicle content disappeared, and the matrix was light; in the RFK(-) group, the matrix of the neurons was light, the synaptic vesicles were extremely dilated, the contents disappeared, the membranes before and after the synaptic vesicles were separated, and the ER was dilated; in the RFK( +) group, the structure of the neurons was basically normal, and the state of the neurons was similar to that of the control group. These results are consistent with those in vivo experimental results and again indicate that RFK(+) has a protective effect on ischemic neurons, whereas RFK(-) has a detrimental effect on ischemic neurons.

### RFK regulates ER proteins

If RFK is an anti-ischemic brain injury protein, how does it work? What is the signaling pathway through which it acts? Through a large number of literature searches, we decided to observe the effects of RFK on endoplasmic reticulum stress (ERS) related proteins, including Ero1, Caspase 12, CHOP (C/EBP homology protein) and Caspase 3.

**Fig. 4:**
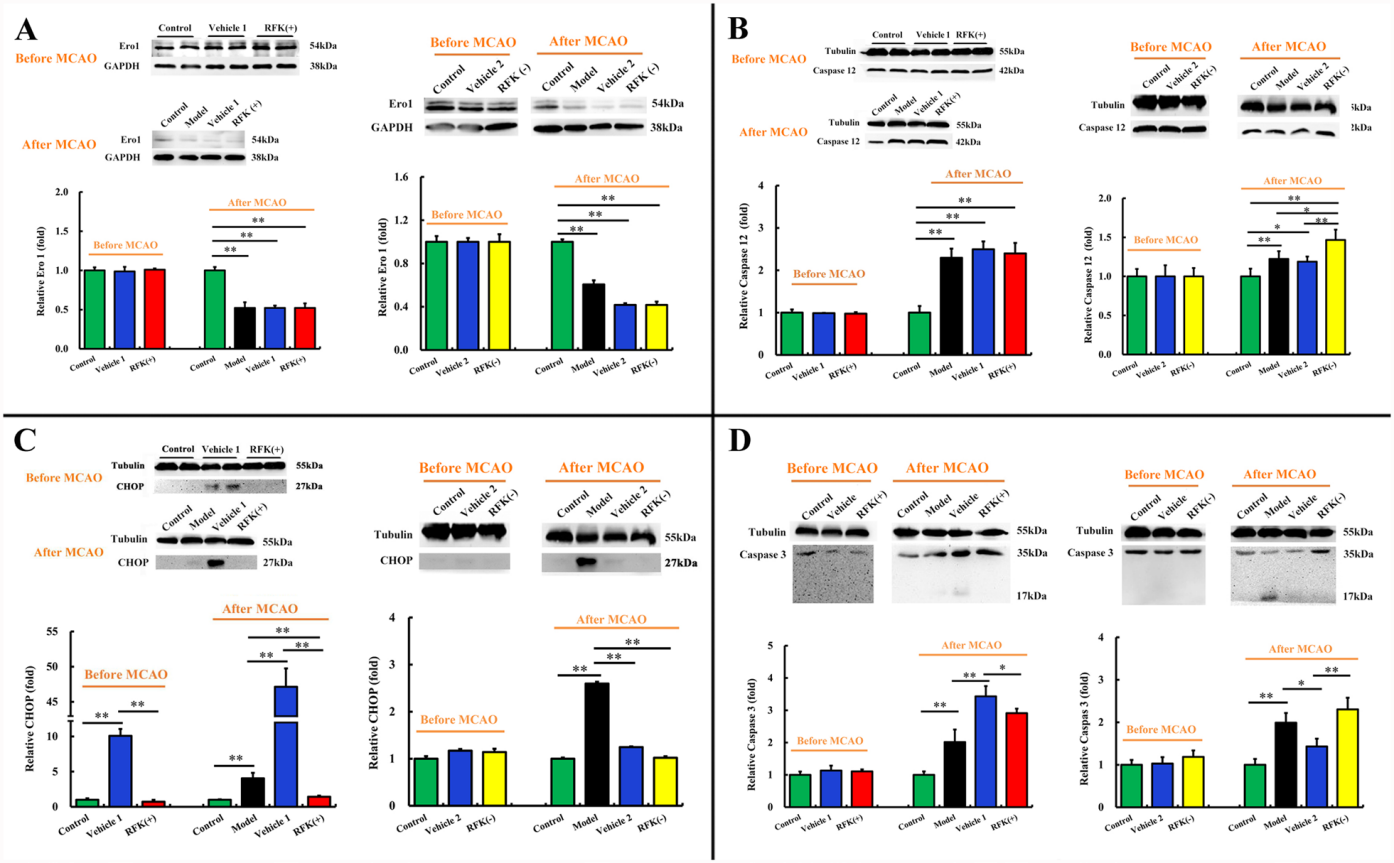
Protein expression levels in rat cortex. Lentiviral vectors encoding RFK knock-up (5 μl per site; 4 × 10^5^TU/µl, empty lentiviral vectors (vehicle) or saline control) were stereotaxically injected into the cortex and hippocampus. Three weeks later, these animals were subjected to MCAO. 24 h later, the rats were anaesthetized and the brains were immediately separated for protein extraction. The protein levels were analyzed by western blotting. **A** Ero1 protein levels in RFK (+) rats (n = 6) and RFK(-) rats (n = 3). **B** Caspase 12 protein level in RFK (+) rats (n = 6) and RFK(-) rats (n = 3). **C** CHOP protein level in RFK( +) rats (n = 6) and RFK(−) rats (n = 3). **D** Caspase 3 protein level in RFK (+) rats (n = 3) and RFK(-) rats (n = 3). Data are presented as mean ± SD. **P < 0.01, *P < 0.05

The cortex protein of rats was extracted to determine the ERS related protein levels. As Fig. 4 shown, without MCAO, the protein levels of Ero1 (Fig. 4A), Caspase 12 (Fig. 4B) and Caspase 3 (Fig. 4D) in the rat cerebral cortex were not affected by RFK (+) or RFK(−). After MCAO, the protein level of Ero1 significantly decreased, and the level of RFK did not change this decrease (*P* < 0.01, Fig. 4A); the protein level of Caspase 12 significantly increased, and RFK(−) aggravated this increase (*P* < 0.01, Fig. 4B); the total protein level of Caspase 3 significantly increased (*P* < 0.01, Fig. 4D); compared with the vehicle group, RFK( +) decreased (*P* < 0.05, Fig. 4D) and RFK(−) increased (*P* < 0.01, Fig. 4D) the total protein level of Caspase 3. Furthermore, the decreased activation fragment of Caspase 3 in the RFK (−) group indicated that RFK( +) inhibited the activation of Caspase 3; and the dramatically increased Caspase 3 zymogen in the RFK(−) group indicated that RFK(-) promoted the gene expression of Caspase 3.

Compared with the control group, the protein level of CHOP in the model group was significantly higher than that in the control group (*P* < 0.01, Fig. 4C). This indicated that the ERS was activated by MCAO in the rat brain. However, no regular effect of RFK on CHOP protein level was observed.

Increased Caspase 12 and CHOP protein expression is a sign of ERS. Overexpression of CHOP (Tajiri et al. 2004) and Caspase 12 (García de la Cadena et al. 2016) promotes cell cycle arrest or apoptosis. The above results suggested that the protein level of Ero1 was downregulated, whereas the protein levels of Caspase 12, CHOP and Caspase 3 were upregulated by MCAO; The RFK(-) aggravated the overexpression of Caspase 12 in ERS induced by MCAO, and thus promoted the high expression of apoptotic protein Caspase 3. However, RFK did not affect the protein level of Ero1 in the rat brain. These indicated that RFK plays an anti-ischemic role in brain injury by inhibiting proteins in the ER stress pathway.

### RFK protection is independent of flavins

To observe the dependence of RFK protection on flavins, we designed the following experiments. Flavins refer to riboflavin, FMN and FAD.

**Fig. 5:**
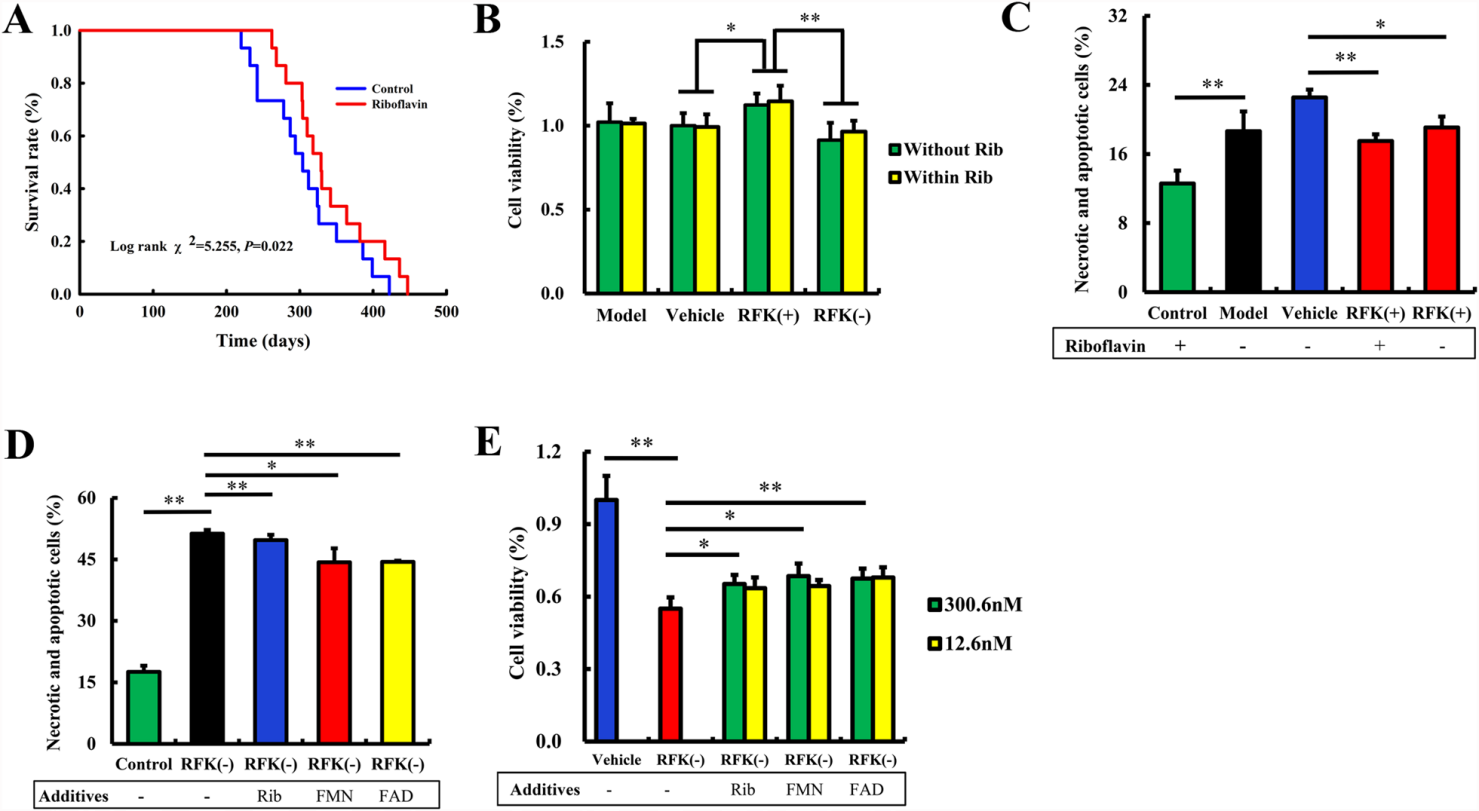
RFK protection doesn’t depend on riboflavin, but riboflavin supplementation probably partially increase the protective effect of RFK. **A** Lifelong supplementation with riboflavin prolonged the lifespan of SHR-SP rats (n = 15). **B**–**C** RFK inhibits damages to neurons induced by OGD (oxygen and glucose deprivation); and the inhibition doesn't depend on the presence of riboflavin. Neurons were infected with empty lentivirus (vehicle), RFK knock-up (RFK( +)) lentivirus and RFK knock-down (RFK(-)) lentivirus for 12 h (MOI = 10). After removing the lentivirus and culturing for another 3 days, the neurons were subjected to the OGD program. Rib: riboflavin. **D**–**E** Supplementation with riboflavin, FMN or FAD partially protects neuron but cannot reverse the damage caused by RFK dysfunction in OGD model. Neuronal apoptosis and death rates were assessed by flow cytometry (n = 3). Neuronal viability was assessed by MTT method (n = 6). Data are presented as mean ± SD. **P < 0.01, *P < 0.05

The lifespan prolonging effect of riboflavin on SHR-SP (which was recognized as RFK gene dysfunction (Zou et al. 2012)) was observed by increasing the daily riboflavin intake (120 mg/L) throughout life, and the compensatory effect of supplemental riboflavin intake on RFK dysfunction was evaluated. As shown in Fig. 5A, the life spans of SHR-SP in the riboflavin group were significantly prolonged compared with the control group (Log rank χ^2^ = 5.255, *P* = 0.022). This result suggested that riboflavin supplementation throughout life can significantly prolong the lifespans of SHR-SP. It also suggested that riboflavin supplementation probably at least partially compensate for the effects of RFK gene dysfunction.

Riboflavin supplementation can alleviate the effects of RFK gene dysfunction, so is the protective effect of RFK dependent on riboflavin?

We then observed the effects of RFK( +) and RFK(−) on neurons exposed to OGD with or without riboflavin. As shown in Fig. 5B, in the absence of riboflavin, the protective effect of RFK( +) on neurons was still present (P < 0.01); in the presence of riboflavin, the protective effect of RFK( +) on neurons showed a trend toward enhancement, but there was no statistical significance; with riboflavin, the neuronal activity of RFK(−) showed a trend toward enhancement compared to that without riboflavin, but the difference was not statistically significant. These results indicated that the absence of riboflavin did not affect the protective effect of RFK, i.e. the protective effect of RFK is independent of riboflavin; however, the presence of riboflavin enhanced the neuronal activity of RFK(+) or RFK(−) to a small extent, suggesting that riboflavin supplementation had a small compensatory effect on RFK dysfunction, but could not completely reverse the damaging effect caused by RFK dysfunction.

Whether RFK affected the tolerance of neurons to OGD in the absence of riboflavin was observed by flow cytometry. As shown in Fig. 5C and Additional file 2, without riboflavin, the necrosis rate of RFK( +) neurons after OGD was still lower than that of the vehicle group (P < 0.01), but the necrosis rate was higher than that of the RFK( +) group with riboflavin (P < 0.05). These results further confirmed that the protective effect of RFK against ischemic injury was not entirely dependent on riboflavin, but its coexistence with riboflavin could enhance the protective effect of RFK. Removing riboflavin showed a trend towards reducing the protective effect of RFK, but there was no statistically significant difference (Fig. 5C, Additional file 2).

Riboflavin plays its role only after it has been catalyzed by RFK into its two active forms, FMN and FAD. Is it because of the RFK gene dysfunction that the conversion of riboflavin is affected and thus cannot play a protective role? This is why we designed the following experiment.

The effect of exogenous flavins supplementation on the decreased neuronal activity of RFK(-) was observed to evaluate the compensatory effect of flavins supplementation on the dysfunction of RFK gene. As shown in Fig. 5D, after OGD, the activity of RFK(-) neurons decreased significantly (P < 0.01), whereas supplementation with riboflavin (P < 0.05), FMN (P < 0.05) and FAD (P < 0. 01) protected neuronal activity to some extent, but the protective effect was not strong and could not completely reverse the neuronal damage caused by RFK(-). And the flavins protective effect on ischemic neurons was independent of their concentration (300.6 nM or 12.6 nM) (Fig. 5E). The concentration of 12.6 nM represents the physical concentration of riboflavin in human plasma; the concentration of 300.6 nM represents the riboflavin concentration in human plasma after riboflavin supplementation.

Therefore, we inferred that the susceptibility to ischemic brain injury induced by RFK gene dysfunction was not related to the reduced conversion of riboflavin to the active forms of FMN and FAD, nor to their concentration.

### Ero1 inhibitor removes RFK protection

**Fig. 6:**
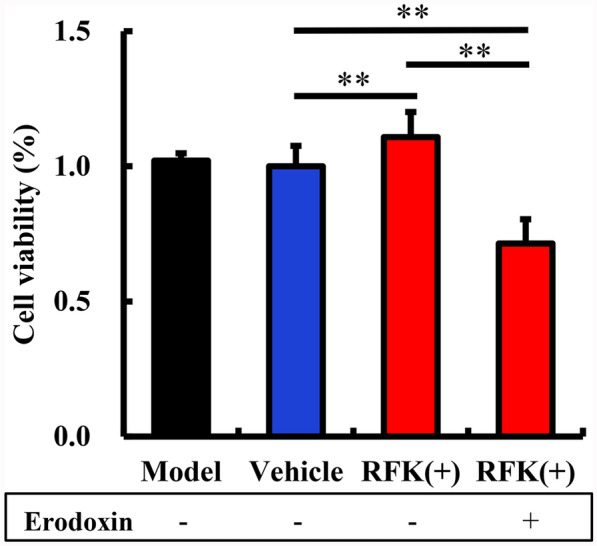
The addition of erodoxin (Ero1 inhibitor) disrupts the protective effect of RFK (n = 6). Neurons were infected with empty lentivirus (vehicle) or RFK knock-up (RFK( +)) lentivirus for 12 h (MOI = 10). After removal of the lentivirus and culturing for a further 3 days, the neurons were subjected to the OGD program. Neuronal viability was assessed by the MTT method. Data are presented as mean ± SD. **P < 0.01, *P < 0.05

Since RFK is an inhibitor of ERS, can the protective effect of RFK on ischemic neurons be blocked by Ero1 inhibitors? To address this question, we observed the tolerance of RFK( +) neurons to OGD by the external addition of erodoxin, an Ero1 inhibitor. As shown in Fig. 6, the protection of RFK (+) was lost. This result indicated that the effect of RFK on ischemic neuronal injury depended on Ero1. It also demonstrated that RFK was involved in the downstream process of Ero1 mediated ERS regulation.

## Discussion

More and more evidence suggest the protective role of RFK in the central nervous system. The RFK gene and FMN alleviate cellular Aβ42 toxicity, which is highly associated with Alzheimer’s disease (Chen et al. 2020). RFK is required for 4, 4’-dimethoxychalcone mediated neuroprotection in Parkinson’s disease (PD) mouse models, and reduction of RFK may be related to PD pathogenesis (Gong et al. 2021). RFK is involved in the regulation of circadian rhythms (Hirano et al. 2017). RFK overexpression abolished the effects of FMN in inhibiting inflammation induced cognitive decline (Zhang et al. 2023). Research on the search for RFK ligands has been reported (Rivero et al. 2023).

Our previous study showed that RFK gene dysfunction may be associated with susceptibility to ischemic stroke (Zou et al. 2012). In this study, the protective role and mechanism of RFK against ischemic stroke were intensively investigated.

Model animal experiments often better reflect the overall effects of target molecules in complex body systems. As this study showed, RFK inhibited the cerebral ischemic injuries induced by MCAO in rats: RFK( +) reduced the infarct size and the brain edema induced by MCAO; RFK ( +) protected, while RFK(−) worsens the subcellular structural damage of the rat cerebral cortex induced by MCAO. These results indicated that RFK inhibited the cerebral ischemic injuries in MCAO rats.

Neurons are the most fundamental structural and functional units of the nervous system, playing a role in connecting, integrating, and transmitting information. Neuron damage caused by ischemic stroke often leads to serious consequences such as disability and motor disorders in patients. Therefore, studying the role of RFK on ischemic damage to neurons is an important aspect of exploring the protective effect of RFK on ischemic brain tissue. In our results, RFK( +) played a protective role in OGD neurons, while RFK(-) exacerbated the injuries in these neurons, including decreasing activity, increasing apoptosis and aggravating ultrastructural damages. These results indicated that RFK inhibited the injury in OGD neurons.

Oxidative stress, calcium metabolism disorders and ischemia–reperfusion injury can all lead to protein misfolding in the ER (Le Pape et al. 2014; Zheng et al. 2015; Krebs et al. 2015). Accumulation of misfolded proteins and concomitant induction of ERS in neurons contributes to neuronal dysfunction (Sprenkle et al. 2017). Regulating ERS significantly inhibited ischemic brain injury (Xin et al. 2014). ERS regulates OGD-induced cell death by ameliorating intracellular ROS (Wang et al. 2018).

To further investigate the mechanism of protective effect of RFK against ischemic brain injury, we observed the effect of RFK on ERS-related proteins, including Ero1, Caspase 12, CHOP and Caspase 3.

The increased expression of CHOP protein is a hallmark of ERS, and overexpression of CHOP promotes cell cycle arrest or apoptosis. In our MCAO model, the CHOP protein was upregulated, suggesting that the ERS was activated in the rat brain. CHOP was reported to mediate the ERS pathway in ischemia-induced neuronal death (Tajiri et al. 2004) and participate in ROS induced neuronal apoptosis (Lu et al. 2014). However, we did not observe a consistent effect of RFK levels on MCAO induced CHOP protein overexpression in our experiment.

Ero1, a member of the flavin protein family, is mainly involved in the oxidative folding of proteins in the ER and mediates ER-associated death (ER-associated death, ERAD) (Li et al. 2009). This process depends on the involvement of FAD (Tu et al. 2000; Tu and Weissman 2002). RFK gene dysfunction will affect the conversion rate of riboflavin in vivo, resulting in a decrease in the concentration of FAD. Ero1 was reported to be a target gene of CHOP (Poone et al 2015). Conversely, there are also reports that CHOP and Ero1 are independent regulators (Marciniak et al. 2004; Varone et al. 2022). In our experiment, the results of increasing CHOP protein and decreasing Ero1 protein in MCAO model more supported the latter theory. It is worth noting that, whether before or after MCAO, RFK does not affect the expression of Ero1 and CHOP. However, erodoxin (Ero1 inhibitor) removed the protective effect of RFK on OGD neurons, suggesting that RFK protection in ischemic neuronal injury depended on Ero1. This further demonstrated that RFK was involved in the downstream signaling regulation of Ero1 in the ERS process.

ERS induced CHOP expression and activated Caspase 12 proteolytic enzymes specific to the ER membrane. CHOP protein is an important intermediate signaling molecule that connects ERS with cell apoptosis, and Caspase 12 is a molecule that executes cell apoptosis. Activated Caspase 12 is transported from the ER to the cytoplasm, activating cytoplasmic Caspase 3 and triggering cell apoptosis. Caspase 12 is involved in the neuronal death of cerebral ischemia (Shibata et al. 2003).

In our MCAO model, RFK did not affect the level of CHOP increasing and Ero1 decreasing; but RFK(-) aggravated the Caspase 12 and Caspase 3 increasing and RFK( +) inhibited the Caspase 3 increasing. These indicated that RFK plays an anti-ischemic role by modulating ER stress pathway; the target molecular might be the upstream factor of Caspase 12; however, CHOP and Ero1 was beyond the modulating scope. RFK( +) decreased and RFK(−) increased the total protein level of Caspase 3. Furthermore, RFK( +) inhibited the activation of Caspase 3 protein; and RFK(−) increased the zymogen amount of Caspase 3. These results further demonstrated the protective effect of RFK in ERS pathway.

Since RFK is a catalytic enzyme for the transformation of riboflavin in the body, the utilization of riboflavin by the body’s cells is first affected by the RFK gene dysfunction; If RFK gene dysfunction increases the susceptibility to stroke (Zou et al. 2012), is it because the decreasing utilization of riboflavin? Can riboflavin supplementing prolong the lifespan of SHR-SP (which was recognized as RFK gene dysfunction)?

Riboflavin is one of the thirteen essential vitamins in the human body, which cannot be synthesized by the body itself and needs to be obtained from food. Riboflavin is a potential neuroprotective agent affecting a wide range of neurological disorders by ameliorating oxidative stress, mitochondrial dysfunction, neuroinflammation, and glutamate excitotoxicity, all of which are implicated in neurological disorders (Marashly et al. 2017). Riboflavin deficiency in rats resulted in reduced levels of myelin lipids, cerebrosides, sphingomyelin and phosphatidylethanolamine in the cerebrum and cerebellum, leading to impaired brain development and maturation (Erecinska et al. 2004). Riboflavin has a good safety record, and excess riboflavin in the body is excreted through the bladder (Buehler et al. 2011). Acute stroke patients were found riboflavin deficient immediately post-infarct (Gariballa et al. 2007). Riboflavin supplements prevented the cognitive decline of elderly adults (Li et al. 2021a, b). Our previous research found that riboflavin pretreatment reduced the apoptosis rate and mortality rate of isolated OGD neurons, suggesting that riboflavin pretreatment may have a protective effect on ischemic brain injury (Zou et al. 2012). Can riboflavin supplementation improve the susceptibility to stroke caused by RFK gene dysfunction and thereby prolong the survival cycle? We selected SHR-SP with RFK gene dysfunction to conduct this experiment.

Riboflavin, through its cofactors FMN and FAD, regulates the structure and function of flavoenzymes and protects cells from oxidative stress and apoptosis (Wanders et al. 2010). Therefore, it is not surprising that any disruption in riboflavin metabolism and absorption may affect cellular levels of FAD and FMN, leading to mitochondrial dysfunction through reduced energy levels (Udhayabanu et al. 2017).

In our experiment on lifespan of SHR-SP and neuronal viability, the presence of flavins probably at least partially compensated for the effect of RFK dysfunction, but could not completely reverse the detrimental effect caused by RFK dysfunction. In our OGD model, the protective effect of RFK against ischemic injury was independent of riboflavin, but the coexistence of riboflavin could enhance the protective effect of RFK. Therefore, we concluded that the susceptibility to ischemic brain injury induced by RFK gene dysfunction was not related to the reduced conversion of riboflavin to the active forms of FMN and FAD, nor to the concentration of flavins; however, the presence of flavins reduced to some extent the negative effect of RFK dysfunction. Conversely, RFK was reported to be inhibited by the reaction products FMN or FAD, but not by substrates riboflavin and ATP (Anoz-Carbonell et al. 2020; Sebastián et al. 2017). However, these conclusions were based on chemical experiments, which may be the main reason why they differ from our conclusions based on cell experiments.

Among the reports on the effect of riboflavin in the pathogenesis of stroke, the interaction between riboflavin and serum homocysteine was mostly followed (Hankey et al. 1999; Frosst et al. 1995); but there has been no report linking the riboflavin protective role in stroke with RFK gene dysfunction. Riboflavin pretreatment reduced the injury induced by ischemia in rats (Hoane et al. 2005; Bets et al. 1994; Mack et al. 1995). The compensatory effect of riboflavin on RFK gene dysfunction may partially explain the effectiveness of riboflavin adjuvant drug therapy after acute stroke (Gariballa et al. 2007; Da Silva‐Candal et al. 2018).

In conclusion, we have demonstrated that RFK is an endogenous protein against ischemic brain injury, and that RFK inhibits the area of infarction, oedema and neuronal apoptosis after cerebral ischemia. Its mechanisms include inhibition of the protein expression of Caspase 12 and Caspase 3 induced by cerebral ischemia, thereby inhibiting ERS and inhibiting neuronal apoptosis; the protective effect of RFK depends on the presence of the flavoprotein Ero1; exogenous riboflavin supplementation protected neurons against ischemic injury and prolonged the lifespan of SHR-SP rats with low RFK gene function, but this protective effect is limited and cannot completely reverse the decreasing trend of neuronal tolerance to ischemic injury caused by RFK gene dysfunction; the protective effect of RFK against ischemic injury is independent of the presence of flavins and their concentrations.

However, there are some limitations to this study. Although we have discovered some signaling molecules in the process of anti-cerebral ischemic injury by RFK, we have not yet identified a regulatory target molecule of RFK. Furthermore, due to species differences, extrapolation of the data to humans should be done with great caution.

RFK deserves further investigation as a promising target gene for stroke susceptibility detection. Flavins can be used as a preventive or adjunctive treatment for ischemic brain injury. They may also be used as partial compensatory medicines for RFK gene dysfunction.

## Supplementary Information

Additional file 1.

Additional file 2.

## Data Availability

No datasets were generated or analysed during the current study.

## Abbreviations

CHOP: C/EBP homology protein
ER: Endoplasmic reticulum
Ero1: Endoplasmic reticulum oxidase 1
ERS: Endoplasmic reticulum stress
FAD: Flavin adenine dinucleotide
FMN: Flavin mononucleotide
MCAO: Middle cerebral artery occlusion
OGD: Oxygen and glucose deprivation
PD: Parkinson’s disease
RFK: Riboflavin kinase/flavokinase
RFK( +): RFK knock-up
RFK(−): RFK knock-down

## References

[CR1] Anoz-Carbonell E, Rivero M, Polo V, Velázquez-Campoy A, Medina M. Human riboflavin kinase: species-specific traits in the biosynthesis of the FMN cofactor. FASEB J. 2020;34(8):10871–86. 32649804 10.1096/fj.202000566R

[CR2] Bets AL, Ren XD, Ennis SR, Hultquist DE. Riboflavin reduces edema in focal cerebral ischemia. Acta Neurochir Suppl (Wien). 1994;60:314–7. 7976577 10.1007/978-3-7091-9334-1_84

[CR3] Buehler BA, Vitamin B. 2: Riboflavin. J Evid-Based Complement Altern Med. 2011;16(2):88–90.

[CR4] Chen X, Ji B, Hao X, Li X, Eisele F, Nyström T, Petranovic D. FMN reduces Amyloid-β toxicity in yeast by regulating redox status and cellular metabolism. Nat Commun. 2020;11(1):867. 32054832 10.1038/s41467-020-14525-4 PMC7018843

[CR5] Da Silva-Candal A, Pérez-Díaz A, Santamaría M, Correa-Paz C, Rodríguez-Yáñez M, Ardá A, Pérez-Mato M, Iglesias-Rey R, Brea J, Azuaje J, Sotelo E, Sobrino T, Loza MI, Castillo J, Campos F. Clinical validation of blood/brain glutamate grabbing in acute ischemic stroke. Ann Neurol. 2018;84(2):260–73. 30014516 10.1002/ana.25286

[CR6] Erecinska M, Cherian S, Silver IA. Energy metabolism in mammalian brain during development. Prog Neurobiol. 2004;73(6):397–445. 15313334 10.1016/j.pneurobio.2004.06.003

[CR7] Frosst P, Blom HJ, Milos R, Goyette P, Sheppard CA, Matthews RG, Boers GJ, den Heijer M, Kluijtmans LA, van den Heuvel LP, et al. A candidate genetic risk factor for vascular disease: a common mutation in methylenetetrahydrofolate reductase. Nat Genet. 1995;10(1):111–3. 7647779 10.1038/ng0595-111

[CR8] García de la Cadena S, Massieu L. Caspases and their role in inflammation and ischemic neuronal death Focus on caspase. Apoptosis. 2016;21(7):763–77. 27142195 10.1007/s10495-016-1247-0

[CR9] Gariballa S, Ullegaddi R. Riboflavin status in acute ischaemic stroke. Eur J Clin Nutr. 2007;61(10):1237–40. 17299470 10.1038/sj.ejcn.1602666

[CR10] Gong J, Zhang W, Ding L, Zhang M, Zheng S, Ma R, Tang J, Yi W, Xu H, Zhang Y. 4,4′-Dimethoxychalcone regulates redox homeostasis by targeting riboflavin metabolism in Parkinson’s disease therapy. Free Radic Biol Med. 2021;174:40–56. 34332078 10.1016/j.freeradbiomed.2021.07.038

[CR11] Guo JM, Liu AJ, Zang P, Dong WZ, Ying L, Wang W, Xu P, Song XR, Cai J, Zhang SQ, Duan JL, Mehta JL, Su DF. ALDH2 protects against stroke by clearing 4-HNE. Cell Res. 2013;23(7):915–30. 23689279 10.1038/cr.2013.69 PMC3698638

[CR12] Hankey GJ, Eikelboom JW. Homocysteine and vascular disease. Lancet. 1999;354(9176):407–13. 10437885 10.1016/S0140-6736(98)11058-9

[CR13] Hilkens NA, Casolla B, Leung TW, de Leeuw FE. Stroke. Lancet. 2024;403(10446):2820–36. 38759664 10.1016/S0140-6736(24)00642-1

[CR14] Hirano A, Braas D, Fu YH, Ptáček LJ. FAD regulates CRYPTOCHROME protein stability and circadian clock in mice. Cell Rep. 2017;19(2):255–66. 28402850 10.1016/j.celrep.2017.03.041 PMC5423466

[CR15] Hoane MR, Wolyniak JG, Akstulewicz SL. Administration of riboflavin improves behavioral outcome and reduces edema formation and glial fibrillary acidic protein expression after traumatic brain injury. J Neurotrauma. 2005;22(10):1112–22. 16238487 10.1089/neu.2005.22.1112

[CR16] Kanazawa H, Shigemoto R, Kawasaki Y, Oinuma KI, Nakamura A, Masuo S, Takaya N. Two-component flavin-dependent riboflavin monooxygenase degrades riboflavin in devosia riboflavina. J Bacteriol. 2018;200(12):e00022-e118. 29610214 10.1128/JB.00022-18 PMC5971479

[CR17] Krebs J, Agellon LB, Michalak M. Ca(2+) homeostasis and endoplasmic reticulum (ER) stress: an integrated view of calcium signaling. Biochem Biophys Res Commun. 2015;460(1):114–21. 25998740 10.1016/j.bbrc.2015.02.004

[CR18] Kuriakose D, Xiao Z. Pathophysiology and treatment of stroke: present status and future perspectives. Int J Mol Sci. 2020;21(20):7609. 33076218 10.3390/ijms21207609 PMC7589849

[CR19] Le Pape S, Dimitrova E, Hannaert P, Konovalov A, Volmer R, Ron D, Thuillier R, Hauet T. Polynomial algebra reveals diverging roles of the unfolded protein response in endothelial cells during ischemia-reperfusion injury. FEBS Lett. 2014;588(17):3062–7. 24945730 10.1016/j.febslet.2014.05.065

[CR20] Li G, Mongillo M, Chin KT, Harding H, Ron D, Marks AR, Tabas I. Role of ERO1-alpha-mediated stimulation of inositol 1,4,5-triphosphate receptor activity in endoplasmic reticulum stress-induced apoptosis. J Cell Biol. 2009;186(6):783–92. 19752026 10.1083/jcb.200904060 PMC2753154

[CR21] Li DJ, Sun SJ, Fu JT, Ouyang SX, Zhao QJ, Su L, Ji QX, Sun DY, Zhu JH, Zhang GY, Ma JW, Lan XT, Zhao Y, Tong J, Li GQ, Shen FM, Wang P. NAD + -boosting therapy alleviates nonalcoholic fatty liver disease via stimulating a novel exerkine Fndc5/irisin. Theranostics. 2021a;11(9):4381–402. 33754067 10.7150/thno.53652 PMC7977447

[CR22] Li S, Guo Y, Men J, Fu H, Xu T. The preventive efficacy of vitamin B supplements on the cognitive decline of elderly adults: a systematic review and meta-analysis. BMC Geriatr. 2021b;21(1):367. 34134667 10.1186/s12877-021-02253-3 PMC8207668

[CR23] Lu TH, Tseng TJ, Su CC, Tang FC, Yen CC, Liu YY, Yang CY, Wu CC, Chen KL, Hung DZ, Chen YW. Arsenic induces reactive oxygen species-caused neuronal cell apoptosis through JNK/ERK-mediated mitochondria-dependent and GRP 78/CHOP-regulated pathways. Toxicol Lett. 2014;224(1):130–40. 24157283 10.1016/j.toxlet.2013.10.013

[CR24] Mack CP, Hultquist DE, Shlafer M. Myocardial flavin reductase and riboflavin: a potential role in decreasing reoxygenation injury. Biochem Biophys Res Commun. 1995;212(1):35–40. 7612015 10.1006/bbrc.1995.1932

[CR25] Maida CD, Norrito RL, Daidone M, Tuttolomondo A, Pinto A. Neuroinflammatory mechanisms in ischemic stroke: focus on cardioembolic stroke, background, and therapeutic approaches. Int J Mol Sci. 2020;21(18):6454. 32899616 10.3390/ijms21186454 PMC7555650

[CR26] Marashly ET, Bohlega SA. Riboflavin has neuroprotective potential: focus on parkinson’s disease and migraine. Front Neurol. 2017;8:333. 28775706 10.3389/fneur.2017.00333 PMC5517396

[CR27] Marciniak SJ, Yun CY, Oyadomari S, Novoa I, Zhang Y, Jungreis R, Nagata K, Harding HP, Ron D. CHOP induces death by promoting protein synthesis and oxidation in the stressed endoplasmic reticulum. Genes Dev. 2004;18(24):3066–77. 15601821 10.1101/gad.1250704 PMC535917

[CR28] Mehrpour S, Rodrigues CR, Ferreira RC, da Briones MR, Oliveira ASB. Hardy-Weinberg Equilibrium in different mitochondrial haplogroups of four genes associated with neuroprotection and neurodegeneration. Arq Neuropsiquiatr. 2020;78(5):269–76. 32490968 10.1590/0004-282x20200002

[CR29] Merrill AH, McCormick DB. Preparation of flavin 5’-phosphates using immobilized flavokinase. Methods Enzymol. 1980;66:287–90. 6246391 10.1016/0076-6879(80)66470-2

[CR30] Poone G, Hasseldam H, Munkholm N, Rasmussen R, Grønberg N, Johansen F. The hypothermic influence on CHOP and Ero1-α in an endoplasmic reticulum stress model of cerebral ischemia. Brain Sci. 2015;5(2):178–87. 25989620 10.3390/brainsci5020178 PMC4493463

[CR31] Rivero M, Boneta S, Novo N, Velázquez-Campoy A, Polo V, Medina M. Riboflavin kinase and pyridoxine 5′-phosphate oxidase complex formation envisages transient interactions for FMN cofactor delivery. Front Mol Biosci. 2023;10:1167348. 37056721 10.3389/fmolb.2023.1167348 PMC10086132

[CR32] Sairanen T, Karjalainen-Lindsberg ML, Paetau A, Ijäs P, Lindsberg PJ. Apoptosis dominant in the periinfarct area of human ischaemic stroke-a possible target of antiapoptotic treatments. Brain J Neurol. 2006;129(Pt 1):189–99. 10.1093/brain/awh645 16272167

[CR33] Sebastián M, Lira-Navarrete E, Serrano A, Marcuello C, Velázquez-Campoy A, Lostao A, Hurtado-Guerrero R, Medina M, Martínez-Júlvez M. The FAD synthetase from the human pathogen Streptococcus pneumoniae: a bifunctional enzyme exhibiting activity-dependent redox requirements. Sci Rep. 2017;7(1):7609. 28790457 10.1038/s41598-017-07716-5 PMC5548840

[CR34] Shan X, Ji Z, Wang B, Zhang Y, Dong H, Jing W, Zhou Y, Hu P, Cui Y, Li Z, Yu S, Zhou J, Wang T, Shen L, Liu Y, Yu Q. Riboflavin kinase binds and activates inducible nitric oxide synthase to reprogram macrophage polarization. Redox Biol. 2024;78: 103413. 39536592 10.1016/j.redox.2024.103413 PMC11605425

[CR35] Shibata M, Hattori H, Sasaki T, Gotoh J, Hamada J, Fukuuchi Y. Activation of caspase-12 by endoplasmic reticulum stress induced by transient middle cerebral artery occlusion in mice. Neuroscience. 2003;118(2):491–9. 12699784 10.1016/s0306-4522(02)00910-7

[CR36] Sprenkle NT, Sims SG, Sánchez CL, Meares GP. Endoplasmic reticulum stress and inflammation in the central nervous system. Mol Neurodegener. 2017;12(1):42. 28545479 10.1186/s13024-017-0183-y PMC5445486

[CR37] Tabet F, Lee S, Zhu W, Levin MG, Toth CL, Cuesta Torres LF, Vinh A, Kim HA, Chu HX, Evans MA, Kuzmich ME, Drummond GR, Remaley AT, Rye KA, Sobey CG, Vickers KC. microRNA-367-3p regulation of GPRC5A is suppressed in ischemic stroke. J Cereb Blood Flow Metab. 2020;40(6):1300–15. 31296130 10.1177/0271678X19858637 PMC7238381

[CR38] Tajiri S, Oyadomari S, Yano S, Morioka M, Gotoh T, Hamada JI, Ushio Y, Mori M. Ischemia-induced neuronal cell death is mediated by the endoplasmic reticulum stress pathway involving CHOP. Cell Death Differ. 2004;11(4):403–15. 14752508 10.1038/sj.cdd.4401365

[CR39] Tu BP, Weissman JS. The FAD- and O(2)-dependent reaction cycle of Ero1-mediated oxidative protein folding in the endoplasmic reticulum. Mol Cell. 2002;10(5):983–94. 12453408 10.1016/s1097-2765(02)00696-2

[CR40] Tu BP, Ho-Schleyer SC, Travers KJ, Weissman JS. Biochemical basis of oxidative protein folding in the endoplasmic reticulum. Science. 2000;290(5496):1571–4. 11090354 10.1126/science.290.5496.1571

[CR41] Udhayabanu T, Manole A, Rajeshwari M, Varalakshmi P, Houlden H, Ashokkumar B. Riboflavin responsive mitochondrial dysfunction in neurodegenerative diseases. J Clin Med. 2017;6(5):52. 28475111 10.3390/jcm6050052 PMC5447943

[CR42] Varone E, Decio A, Barbera MC, Bolis M, Di Rito L, Pisati F, Giavazzi R, Zito E. Endoplasmic reticulum oxidoreductin 1-alpha deficiency and activation of protein translation synergistically impair breast tumour resilience. Br J Pharmacol. 2022;179(23):5180–95. 35853086 10.1111/bph.15927 PMC9804893

[CR43] Wanders RJA, Ruiter JPN, IJLst L, Waterham HR, Houten SM. The enzymology of mitochondrial fatty acid beta-oxidation and its application to follow-up analysis of positive neonatal screening results. J Inherit Metab Dis. 2010;33(5):479–94. 20490924 10.1007/s10545-010-9104-8 PMC2946543

[CR44] Wang HF, Wang ZQ, Ding Y, Piao MH, Feng CS, Chi GF, Luo YN, Ge PF. Endoplasmic reticulum stress regulates oxygen-glucose deprivation-induced parthanatos in human SH-SY5Y cells via improvement of intracellular ROS. CNS Neurosci Ther. 2018;24(1):29–38. 29045036 10.1111/cns.12771 PMC6490059

[CR45] Wang CQ, Li YT, Zhang YG, Semrin D, Gu LJ, Jiang ST, Xiong XX. Triolein alleviates ischemic stroke brain injury by regulating autophagy and inflammation through the AKT/mTOR signaling pathway. Mol Med. 2024;30(1):242. 39639187 10.1186/s10020-024-00995-5 PMC11622655

[CR46] Xin Q, Ji B, Cheng B, Wang C, Liu H, Chen X, Chen J, Bai B. Endoplasmic reticulum stress in cerebral ischemia. Neurochem Int. 2014;68:18–27. 24560721 10.1016/j.neuint.2014.02.001

[CR47] Yazdanpanah B, Wiegmann K, Tchikov V, Krut O, Pongratz C, Schramm M, Kleinridders A, Wunderlich T, Kashkar H, Utermöhlen O, Brüning JC, Schütze S, Krönke M. Riboflavin kinase couples TNF receptor 1 to NADPH oxidase. Nature. 2009;460(7259):1159–63. 19641494 10.1038/nature08206

[CR48] Zhang M, Chen H, Zhang W, Liu Y, Ding L, Gong J, Ma R, Zheng S, Zhang Y. Biomimetic remodeling of microglial riboflavin metabolism ameliorates cognitive impairment by modulating neuroinflammation. Adv Sci. 2023;10(12):2300180. 10.1002/advs.202300180 PMC10131853 36799538

[CR49] Zheng XY, Xu J, Chen XI, Li W, Wang TY. Attenuation of oxygen fluctuation-induced endoplasmic reticulum stress in human lens epithelial cells. Exp Ther Med. 2015;10(5):1883–7. 26640566 10.3892/etm.2015.2725 PMC4665140

[CR50] Zhou ZY, Li WL, Ni L, Wang TL, Huang Y, Yu YQ, Hu MX, Liu YL, Wang JE, Huang XF, Wang YY. Icariin improves oxidative stress injury during ischemic stroke via inhibiting mPTP opening. Mol Med. 2024;30(1):77. 38840035 10.1186/s10020-024-00847-2 PMC11155182

[CR51] Zou Y, Zhang X, Su F, Liu X. Importance of riboflavin kinase in the pathogenesis of stroke. CNS Neurosci Ther. 2012;18(10):834–40. 22925047 10.1111/j.1755-5949.2012.00379.x PMC6493343

