# Supplementary figures and images for: Neuroprotective effect of riboflavin kinase on cerebral ischemia injury in rats
Source: Mol Med. 2025 Apr 2;31:125. doi: 10.1186/s10020-025-01170-0 (PMC11966939; doi:10.1186/s10020-025-01170-0)

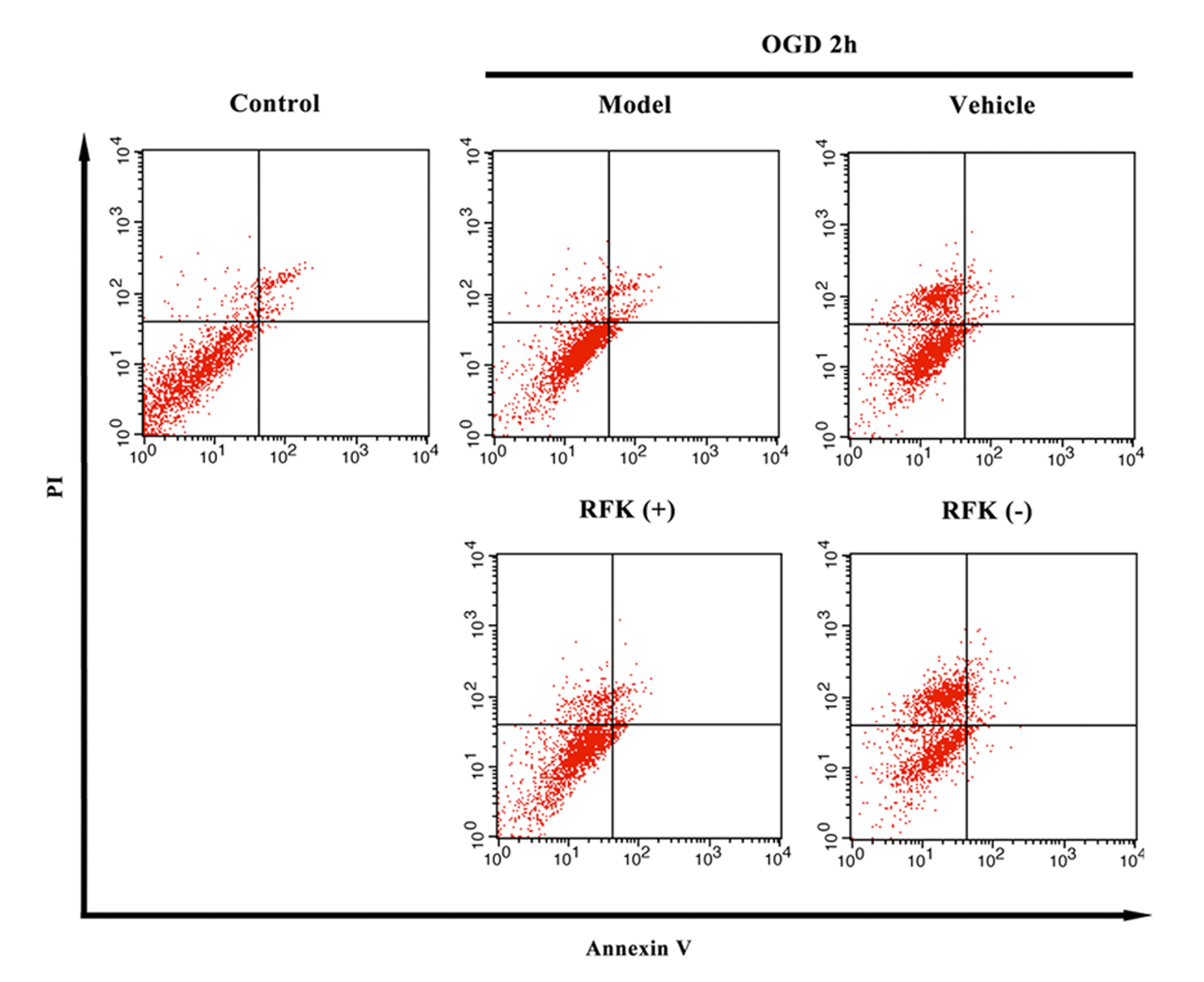

Supplement: Supplementary file 1 — Additional file 1. [file 10020_2025_1170_MOESM1_ESM.tif]

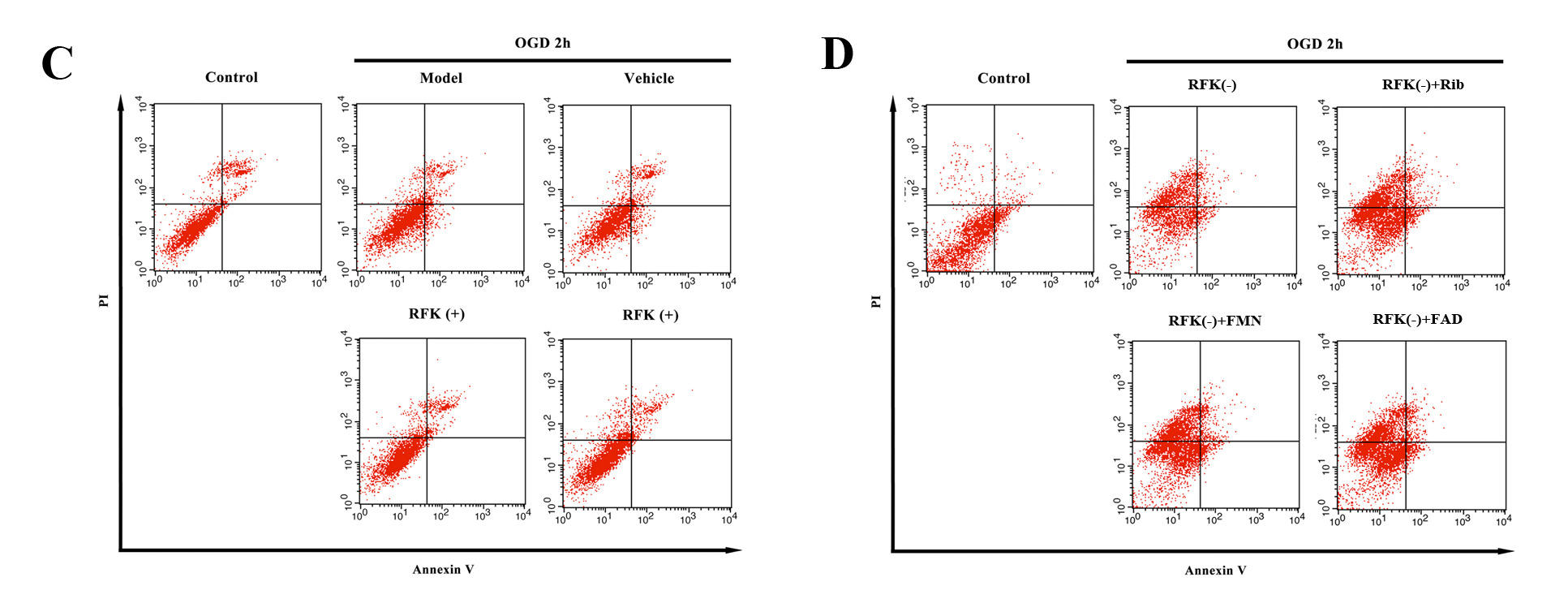

Supplement: Supplementary file 2 — Additional file 2. [file 10020_2025_1170_MOESM2_ESM.tif]
